# CSP 40 years: maturity

**DOI:** 10.1590/0102-311XEN220923

**Published:** 2024-01-08

**Authors:** Marilia Sá Carvalho, Luciana Dias de Lima, Luciana Correia Alves

**Affiliations:** 1 Programa de Computação Científica, Fundação Oswaldo Cruz, Rio de Janeiro, Brasil.; 2 Escola Nacional de Saúde Pública Sergio Arouca, Fundação Oswaldo Cruz, Rio de Janeiro, Brasil.; 3 Instituto de Filosofia e Ciências Humanas, Universidade Estadual de Campinas, Campinas, Brasil.

CSP turns 40 in 2024. It is time to celebrate the maturity the journal has acquired. CSP is, as we stated seven years ago, a common good of Public Health ^1^.

We, the Editors and the technical team, are preparing a year of celebration. It will include events, a label, interviews, themed editorials, a visual exhibition, and much more. This is certainly a special moment to reflect on our journey so far and think about the future.

In this process of reflection, we intend to analyze the profile of the articles published by CSP over time. The objective is to understand which themes have been relevant in this journey and what their impact has been, not only by analyzing the usual bibliometric indicators, but also by incorporating a qualitative view of the contribution of the various sub-fields of knowledge represented in the journal.

In 2024 we will also deepen the debate on open science. Open science is an international movement that aims to make research accessible to all, eliminating artificial obstacles, especially editorial, legal and economic ones, to allow for free circulation of scientific knowledge, as open as possible, and as closed as necessary ^2^. Regarding open access, CSP is one of the few journals that maintain the diamond standard of scientific publication: there is no submission or publication fee, nor is there any access charge for reading the articles. This is only possible thanks to the funding provided by the sponsoring institution, the Sergio Arouca National School of Public Health, Oswaldo Cruz Foundation (ENSP/Fiocruz).

A step towards opening up the evaluation process has already been taken, as the Associated Editors responsible for the article will now be identified. But we must go further. We need to know what the Public Health community in Brazil thinks about open peer review and the sharing of databases, codes, methods, and other materials used in scientific research. For this, we will use a websurvey to invite authors, consultants, and editors to opine on open science publishing practices. This knowledge will be essential for us to move appropriately in this direction.

The CSP technical team is a key element in the quality of the journal. The diversity, qualification and excellence of this group marks CSP’s history. The most recent investment was the hiring of a journalist specialized in science, which made it possible to expand the activity of science communication, an essential aspect of the dissemination of knowledge today. And we need to make even more progress in coordinating CSP with media outlets, in dialoguing with society and decision-makers, and in involving editors and authors in the dissemination of the results of published studies.

Thus, 2024 will be a year of hard work and many challenges. And, of course, a year of celebration. We invite authors and consultants, who make up the CSP community, to share this moment with us.

Happy anniversary! May CSP always be present in the life of Public Health.
